# A Novel Adaptive Time-Frequency Filtering Approach to Enhance the Ultrasonic Inspection of Stainless Steel Structures

**DOI:** 10.3390/s23021030

**Published:** 2023-01-16

**Authors:** Biao Wu, Haitao Yang, Yong Huang, Wensong Zhou, Xiaohui Liu

**Affiliations:** 1 College of Civil Engineering, Nanjing Tech University, Nanjing 211816, China; 2 Key Laboratory of Structural Dynamic Behavior and Control of the Ministry of Education, School of Civil Engineering, Harbin Institute of Technology, Harbin 150090, China; 3 Institute of Engineering Mechanics, China Earthquake Administration, Langfang 065201, China

**Keywords:** stainless steel, ultrasonic nondestructive testing, grain noise, noise suppression, time-frequency filtering

## Abstract

Ultrasonic nondestructive testing (NDT) provides a valuable insight into the integrity of stainless steel structures, but the noise caused by the scattering of stainless steel microstructure often limits the effectiveness of inspection. This work presents a novel adaptive filtering approach to enhance the signal-to-noise ratio (SNR) of a measured ultrasonic signal from the inspection of a stainless steel component, enabling the detection of hidden flaws under strong noise. After the spectral modeling of the noisy ultrasonic NDT signal, the difference between the spectral characteristics of a flaw echo and that of grain noise is highlighted, and a reference spectrum model to estimate the frequency spectrum of the echo reflected by any possible flaw is developed. Then, the signal is segmented and the similarity between the spectra of data segments and the reference spectra is evaluated quantitatively by the spectral similarity index (SSI). Based on this index, an adaptive time-frequency filtering scheme is proposed. Each data segment is processed by the filtering to suppress the energy of noise. The processed data segments are recombined to generate the de-noised signal after multiplying weighting coefficients, which again is determined by the SSI. The performance of the proposed method for SNR enhancement is evaluated by both the simulated and experimental signal and the effectiveness has been successfully demonstrated.

## 1. Introduction

Excellent mechanical properties such as toughness, high strength as well as resistance to corrosion and heat have made stainless steel important building material widely applied in civil and marine engineering. For example, introducing stainless steel tubes to replace the carbon steel tube in concrete-filled-steel-tube systems can fundamentally solve the corrosion problem [1]. Stainless steel is also used as reinforcement for concrete structures in the coastal environment [2] as well as storage canisters and piping systems in the nuclear industry [3]. During long-term service, properties of stainless steel such as strength, ductility and corrosion resistance deteriorate due to aging [4]. Among the factors responsible for the performance deterioration, chloride-induced stress corrosion cracking of welded heat affected zones is of special concern as a well-documented mode of attack for stainless steels [5]. Manufacturing flaws (inclusions, voids or lack of fusion) are another factor of performance deterioration that could also exist in the welded joints. To ensure the safety and integrity of stainless steel structures, ultrasonic nondestructive testing techniques would be very helpful to detect, locate and size potential in-service cracks and manufacturing flaws [6,7]. Especially, state-of-the-art additive manufacturing (AM) requires advanced ultrasonic NDT techniques to evaluate the integrity of AM components [8] and to design an inspection scheme based on the reliability and cost analysis of AM components [9]. 

However, stainless steels are coarse-grained, heterogeneous and anisotropic. The interaction between incident ultrasonic waves and the grain microstructure of stainless steel results in a type of complex acoustic noise, often called grain noise, leading to low SNR of the measured signal and poor detectability of flaws. The instrumental noise of the NDT system could further lower the SNR.

For the effective inspection of stainless steel, SNR enhancement of the measured ultrasonic signal is of great importance, and numerous methods have been proposed for it. Widely explored methods include wavelet-based processing [10,11,12,13], split spectrum processing [14,15,16] and deconvolution-based approaches [17,18,19]. In more recent years, efforts based on sparse signal representation [20,21,22] and empirical mode decomposition [19] also contributed to the enhancement of SNR for ultrasonic NDT.

When an ultrasonic pulse propagates inside a stainless steel component, the pulse will interact with the grain microstructure, resulting in the distortion of the pulse due to scattering and attenuation. In stainless steel, as reported in [23], the ratio of the wavelength λ to the average grain diameter $\bar{D}$ satisfies that $\lambda /\bar{D}\gg 1$. The scattering of the incident ultrasonic wave by the grain microstructure falls in the Rayleigh scattering region where attenuation is proportional to the fourth power of frequency. As a result, the high-frequency component of the ultrasonic wave attenuates more significantly than the low-frequency component, leading to the waveform distortion of the ultrasonic pulse that the center frequency decreases while the duration dilates. Meanwhile, the interaction between the incident ultrasonic pulse and a flaw is different from that of a grain. Generally, the size of a flaw is comparable or larger than the ultrasonic wavelength λ and thus can be viewed as a geometrical reflector, and the reflected signal can be regarded as frequency independent [24]. On the contrary, the backscattering of the incident ultrasonic pulse by a grain is frequency dependent on the amplitude of scattering being proportional to the second power of frequency [25]. The difference between the two interaction mechanisms can be useful for noise suppression in the ultrasonic NDT of stainless steel structures.

Based on the above analysis, this work presents a novel methodology to enhance the ultrasonic NDT of stainless steel materials by exploiting the time-frequency characteristics of the ultrasonic signal. Specifically, the commonly used pulse-echo testing mode will be considered here. It is important to note that the echo reflected by a defect has a finite-time duration and the distortion of the waveform is dependent on the traveling distance. Taking these characteristics into consideration, it is desirable to exploit the time-frequency features of the signal rather than analyzing the signal as a whole. We start with the spectral modeling of grain noise and a typical flaw echo, and how the two spectra evolve with traveling distance is established. The difference between the spectral characteristics of a flaw echo and that of noise is highlighted. A reference spectrum model based on this difference is developed for the echo reflected by any possible flaw. Next, the signal is segmented and the similarity between the spectra of data segments and the corresponding reference spectra is evaluated quantitatively by a term defined as the spectral similarity index (SSI) in a moving window fashion. For each data segment, its SSI will determine the shape of the filter as well as the weighting coefficient which is used in the recombination stage where all the filtered data segments are recombined to generate the de-noised signal.

The rest of this paper is organized as follows. In Section 2, detailed spectral analysis and modeling of the components of a typical ultrasonic NDT signal are conducted. In Section 3, the proposed method is presented in detail. In Section 4, we demonstrate our method by synthetic ultrasonic signals and signals from an experimental ultrasonic inspection of a stainless steel block that contains prefabricated flaws, respectively. In the final section, some conclusive remarks are drawn.

## 2. Modeling of Ultrasonic NDT Signal

When conducting ultrasonic NDT on a stainless steel component in pulse-echo mode, the measured signal $y\left(t\right)$ can be written as $y\left(t\right)=e\left(t\right)+g\left(t\right)+w\left(t\right)$ where $e\left(t\right)$ refers to the echoes reflected by defects in the component, $g\left(t\right)$ and $w\left(t\right)$ are backscattered grain noise and electrical noise caused by the instruments, respectively. 

In pulse-echo mode where the transducer is functioning as both a transmitter and a receiver, the scattered wave by a grain scatterer in the time domain can be viewed as the convolution of the impulse response of a grain scatterer $r\left(t,\lambda \right)$ and the impinging ultrasonic wave $h\left(t,\lambda \right)$ on the scatterer:

(1) $$g\left(t\right)=h\left(t,\lambda \right)*r\left(t,\lambda \right)$$

where ∗ denotes the convolution operator. The impulse response function $r\left(t,\lambda \right)$ mathematically describes the backscattered wave as a function of wavelength λ (or equivalently, frequency) in the time domain. Meanwhile, the impinging ultrasonic pulse $h\left(t,\lambda \right)$ is a convolution of the transmitted pulse from the transducer $u\left(t,\lambda \right)$ and the attenuation function $a\left(t,\lambda \right)$, since the transmitted pulse will experience frequency-dependent attenuation during its propagation from the transmitter to the scatterer. Therefore, the backscattered signal by a grain scatterer received by the receiver can be expressed as:

(2) $$g\left(t\right)=u\left(t,\lambda \right)*a\left(t,\lambda \right)*r\left(t,\lambda \right)$$

In the frequency domain, the above expression can be written as:

(3) $$G\left(f\right)\propto \left|U\left(f\right)\right|\left|S\left(f\right)\right|\left|R\left(f\right)\right|\left|A\left(f\right)\right|$$

where $\left|U\left(f\right)\right|$ is the impulse response of the transducer which is usually modeled as a bandpass Gaussian-shaped spectrum. $\left|S\left(f\right)\right|$ is the frequency modulation function due to the sum of grain scatterers with random orientations and phases [26]. $\left|R\left(f\right)\right|$ is the frequency-dependent scattering function which is proportional to the second power of frequency in the Rayleigh scattering region. $\left|A\left(f\right)\right|$ is the transfer function corresponding to the frequency-dependent attenuation characteristics of the ultrasonic wave during its propagation.

Similar to grain noise, the echo reflected by a flaw can be viewed as a convolution between the impinging ultrasonic pulse and the impulse response function of a flaw, considering the effect of frequency-dependent attenuation:

(4) $$e\left(t\right)=u\left(t,\lambda \right)*a\left(t,\lambda \right)*p_{f}$$

where $p_{f}$ is the impulse response function of a flaw and is independent on frequency. In the frequency domain, a flaw echo can be expressed as:

(5) $$E\left(f\right)\propto \left|U\left(f\right)\right|\left|A\left(f\right)\right|$$

By comparing Equations (3) and (5), it is obvious that the spectrum of a flaw differs from that of grain noise, represented by $\left|S\left(f\right)\right|$ and $\left|R\left(f\right)\right|$. The inherent randomness property of $\left|S\left(f\right)\right|$ will make the spectrum of grain noise take on a grass-like pattern; while $\left|R\left(f\right)\right|$ means that the spectrum of grain noise shifts upward compared to the spectrum of a flaw, due to the frequency dependence scattering property of $\left|R\left(f\right)\right|$ which is proportional to the second power of frequency in the Rayleigh scattering region.

From Equation (5), it is seen that once the attenuation function $A\left(f\right)$ is determined, one can estimate the frequency spectrum of a flaw echo at any given depth. According to earlier studies, the attenuation function can be written as [23,24]:

(6) $$A\left(f\right)\propto exp[-2\int_{0}^{z}\alpha \left(z,f\right)dz]=exp\{-2\int_{0}^{z}\left[\alpha_{a}\left(z,f\right)+\alpha_{s}\left(z,f\right)\right]dz\}$$

where z is the distance of a reflector from the transducer. $\alpha \left(z,f\right)$ is the frequency-dependent attenuation coefficient defined as the sum the scattering term $\alpha_{s}\left(z,f\right)$ and the absorption term $\alpha_{a}\left(z,f\right)$. In the Rayleigh scattering region, the former varies with the third power of grain diameter and the fourth power of frequency while the latter increases linearly with frequency. However, the absorption term is usually insignificant and negligible when compared to the scattering term; therefore, the attenuation model can be expressed as:

(7) $$A\left(f,t\right)=exp[-\alpha \left(2\pi f{)}^{4}vt\right]$$

where v is the velocity of the sound in stainless steel and t is the time delay of the pulse. In practice, the attenuation coefficient α can be estimated beforehand [27]. Generally, the spectrum of the transmitted pulse $U\left(f\right)$ possesses a Gaussian shape with the following form:

(8) $$U\left(f\right)=exp[-\frac{{\left(f-f_{c}\right)}^{2}}{2s^{2}}]$$

where $f_{c}$ is the center frequency of the transmitted pulse, s is a parameter that describes the frequency bandwidth of the transmitted pulse. Combining Equations (7) and (8), the spectrum of a flaw echo can be estimated as:

(9) $$E\left(f\right)=exp[-\frac{{\left(f-f_{c}\right)}^{2}}{2s^{2}}]exp[-\alpha (2\pi f{)}^{4}vt]$$

## 3. The Proposed Methodology

**Figure 1:**
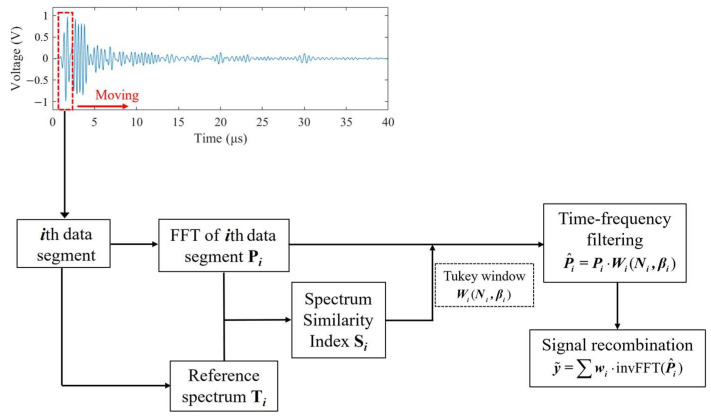
Illustration of the proposed method.

The procedure of the proposed method is illustrated in Figure 1. First, the measured signal is segmented successively along the time axis at a step of one sample. Here, the segment length is set to be slightly longer than the duration of the transmitted pulse because the duration of the pulse increases during propagation due to frequency-dependent attenuation, which results in the decrease of the frequency bandwidth of the pulse. Next, a fast Fourier transform (FFT) operation is performed on the *i*th segmented data of which the time delay is $t_{i}$, after multiplying a Hamming window function, to extract its spectrum $P_{i}$. At the same time, the reference spectrum $T_{i}$ is obtained by substituting $t_{i}$ into Equation (9). The similarity between $P_{i}$ and $T_{i}$ is calculated, yielding the SSI value $S_{i}$. For each data segment, based on its $S_{i}$, a time-frequency filter can be designed employing the Tukey window. The filter is adaptive because, for each data segment, one filter is specifically tailored whose width and shape are determined by the spectral characteristics of the data segment. The spectrum $P_{i}$ is then fed to the filter to produce the spectrum of the de-noised data segment. After inverse FFT operation and recombination, the final de-noised signal can be obtained. The calculation of SSI and the designing of the filter for each data segment are elaborated as follows.

### 3.1. Calculation of Spectral Similarity Index

To evaluate the degree of similarity between $P_{i}$ and $T_{i}$, an appropriate similarity measure is designed first. The correlation coefficient is one of the most used similarity measures. For the two vectors $P_{i}$ and $T_{i}$, the correlation coefficient is calculated by:

(10) $$C=\frac{P_{i}\times T_{i}}{\sqrt{\|P_{i}\|{}^{2}\cdot \|T_{i}\|{}^{2}}}=\frac{\langle P_{i},T_{i}\rangle }{\sqrt{\langle P_{i},P_{i}\rangle \cdot \langle T_{i},T_{i}\rangle }}$$

where $\langle T_{i},P_{i}\rangle =T_{i}{}^{T}\cdot P_{i}$ is the inner product of $P_{i}$ and $T_{i}$. It is worthwhile noting that the calculation of the correlation coefficient is a linear operation and is appropriate for data that are best described by second-order statistics. To evaluate the similarity between two spectra that have complex patterns, second-order statistics may not be sufficient. As reported in [28], by mapping the two variables into high-dimensional space before calculating the correlation coefficient, high-order statistics of the variables can be exploited, thus better performance can be achieved. In this study, a nonlinear technique employing a kernel function is used in the evaluation of similarity between $P_{i}$ and $T_{i}$. First, both $P_{i}$ and $T_{i}$ are nonlinearly mapped into a potentially higher dimensional space, denoted as ${\tilde{P}}_{i}=\Phi \left(P_{i}\right)$ and ${\tilde{T}}_{i}=\Phi \left(T_{i}\right)$, respectively. With the help of a kernel function, one can compute the inner product in the high dimensional space without explicitly computing the nonlinear mapping Φ. 

A kernel function κ computes the inner product of mapped $P_{i}$ and $T_{i}$ as follows:

(11) $$\kappa \left(P_{i},T_{i}\right)=\langle \Phi \left(P_{i}\right),\Phi \left(T_{i}\right)\rangle$$

Substituting normalized variables into Equation (11), one has

(12) $$\tilde{\kappa }\left(P_{i},T_{i}\right)=\langle \frac{\Phi \left(P_{i}\right)}{\left\|\Phi \left(P_{i}\right)\right\|},\frac{\Phi \left(T_{i}\right)}{\left\|\Phi \left(T_{i}\right)\right\|}\rangle =\frac{\kappa \left(P_{i},T_{i}\right)}{\sqrt{\kappa \left(P_{i},P_{i}\right)\cdot \kappa \left(T_{i},T_{i}\right)}}$$

From Equations (10)–(12), it is easily noticed that Equation (12) is equivalent to computing the correlation coefficient between $\Phi \left(P_{i}\right)$ and $\Phi \left(T_{i}\right)$. In this study, a Gaussian kernel function is employed which has the following form:

(13) $$\kappa \left(P_{i},T_{i}\right)=exp(-\frac{\|P_{i}-T_{i}\|{}^{2}}{2\sigma^{2}})$$

where σ>0 is a parameter that controls the flexibility of the kernel. Note that the denominator in Equation (13) equals 1. Therefore, the SSI between $P_{i}$ and $T_{i}$ can be calculated by

(14) $$S_{i}=\tilde{\kappa }\left(P_{i},T_{i}\right)=exp(-\frac{\|P_{i}-T_{i}\|{}^{2}}{2\sigma^{2}})$$

Note that the calculation of SSI above combines two commonly used similarity criteria, i.e., the correlation coefficient and the sum of squares of deviations.

### 3.2. SSI-Ssi-Based Adaptive Time-Frequency Filtering

**Figure 2:**
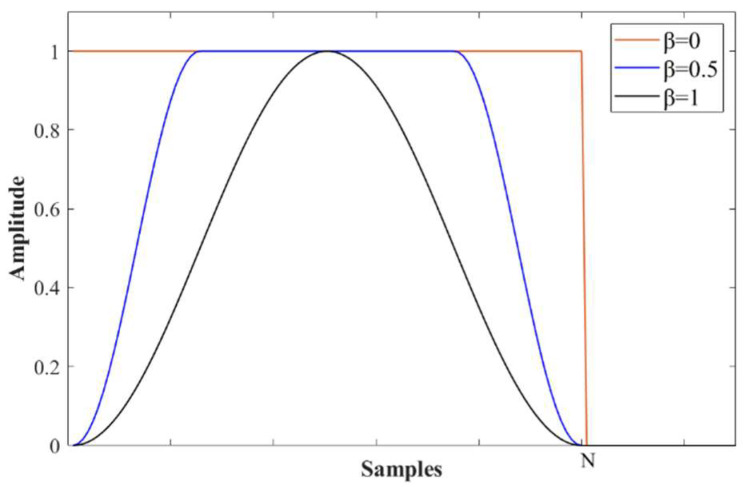
Shapes of Tukey window with various β values.

Essentially, the reference spectrum $T_{i}$ estimates the frequency band and spectrum shape of any possible flaw echo of time delay $t_{i}$. Therefore, a time-frequency filtering scheme can be devised based on the SSI calculated above, which evaluates the similarity between the spectrum of the *i*th data segment and its corresponding reference spectrum. The proposed time-frequency filtering is expressed as follows:

(15) $${\hat{P}}_{i}=P_{i}\cdot W_{i}\left(N_{i},\beta_{i}\right)$$

where ${\hat{P}}_{i}$ is the frequency spectrum of the *i*th data segment after the filtering process, $W_{i}\left(N_{i},\beta_{i}\right)$ is the Tukey window function that has the following form [29]:

(16) $$W\left(n\right)=\left\{\begin{array}{cc} \frac{1}{2}[1-\cos(\frac{2\pi n}{\beta N})], & 0\leq n\leq \frac{\beta N}{2}\\ 1, & \frac{\beta N}{2}\leq n\leq \frac{N}{2}\\ W\left(N-n\right), & 0\leq n\leq N \end{array}\right.$$

where N is the width of the Tukey window, β is a flexible parameter that controls the shape of the window so that at β=0 the window becomes rectangular and at β=1 the window becomes a Hann window. The relation between window shape and β is illustrated in Figure 2.

Because the spectrum of a flaw echo is assumed to process a Gaussian shape where the energy of the signal is distributed in a finite bandwidth centered at the center frequency, the width of the Tukey window N can be determined by the bandwidth of significant energy distribution, which is set to be the −20 dB bandwidth in this study. For example, for the data segment centered at $t_{i}$, its corresponding reference spectrum $T_{i}$ is estimated using Equation (9), the width of the Tukey window $N_{i}$ for this data segment is equal to the −20 dB bandwidth of the reference spectrum, and the shape parameter is determined as $\beta_{i}=1-S_{i}$.

From Equation (15), it can be observed that if the spectrum of a data segment $P_{i}$ highly resembles its reference spectrum $T_{i}$, the SSI between the two spectra will approach 1, the Tukey window is almost rectangular and the designed filter is equivalent to a band-pass filter and has no impact on $P_{i}$. Conversely, if $P_{i}$ is very different from $T_{i}$, the SSI between the two spectra will be near 0, the Tukey window is now a Hann window that modulates the spectrum $P_{i}$ so that the power of signal outside the main frequency band will be reduced to zero and at the meantime keeps remained power concentrated at the predicted center frequency and decayed toward both sides. By doing this, the power of noise is significantly reduced.

After the filtering stage, inverse FFT is applied to ${\hat{P}}_{i}$, and the final de-noised signal can be obtained by the recombination process:

(17) $$\tilde{y}=\sum w_{i}\cdot \mathrm{invFFT}({\hat{P}}_{i})$$

where $w_{i}$ is a weighting coefficient which is determined by SSI as follows:

(18) $$w_{i}=\left\{\begin{array}{cc} 1, & \mathrm{if}\;S_{i}\geq thres\\ 0, & \mathrm{if}\;else \end{array}\right.$$

where thres is the threshold value to dismiss the data segments having insignificant SSI values. In this study, $thres=0.3max(S_{i})$.

The implementation of the proposed method is summarized in Algorithm 1.
**Algorithm 1.** Procedure of the proposed method. **Input:** noisy signal y**Output:** de-noised signal $\tilde{y}$
**Step 1: Data segmentation.**Successive segmentation of the signal, such that:(1) Segment length L is slightly larger than the length of transmitted pulse;(2) The overlapped length of adjacent segments is L−1.**Step 2: Filtering of each data segment.**For the *i*th segment whose time center is $t_{i}$:(1) Calculate its frequency spectrum $P_{i}$ by FFT;(2) Obtain the reference spectrum: $T_{i}=exp[-\frac{{\left(f-f_{c}\right)}^{2}}{2s^{2}}]exp[-\alpha \left(2\pi f{)}^{4}vt_{i}\right]$;(3) Calculate its SSI value $S_{i}$: $S_{i}=\tilde{\kappa }\left(P_{i},T_{i}\right)=exp(-\frac{\|P_{i}-T_{i}\|{}^{2}}{2\sigma^{2}})$;(4) Let $\beta =1-S_{i}$, calculate the Tukey window:

$\;\;\;\;\;\;\;\;W\left(n\right)=\left\{\begin{array}{cc} \frac{1}{2}[1-\cos(\frac{2\pi n}{\beta N})], & 0\leq n\leq \frac{\beta N}{2}\\ 1, & \frac{\beta N}{2}\leq n\leq \frac{N}{2}\\ W\left(N-n\right), & 0\leq n\leq N \end{array}\right.$(5) Calculate the filtered spectrum ${\hat{P}}_{i}$ by: ${\hat{P}}_{i}=P_{i}\cdot W_{i}\left(N_{i},\beta_{i}\right)$;(6) Calculate the weighting coefficient $w_{i}$: $w_{i}=\left\{\begin{array}{cc} 1, & \mathrm{if}\;S_{i}\geq thres\\ 0, & \mathrm{if}\;else \end{array}\right.$.**Step 3: Signal reconstruction.**Reconstruct the de-noised signal $\tilde{y}$: $\tilde{y}=\sum w_{i}\cdot \mathrm{invFFT}({\hat{P}}_{i})$.

## 4. Results

### 4.1. Simulated Signals

**Figure 3:**
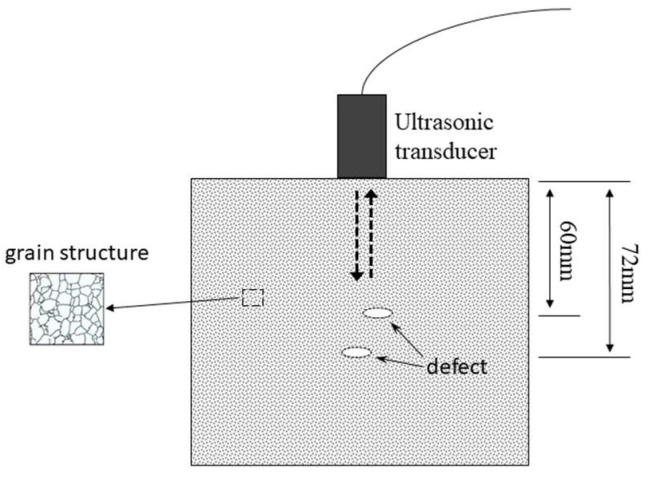
The ultrasonic inspection scenario for the simulation study.

**Figure 4:**
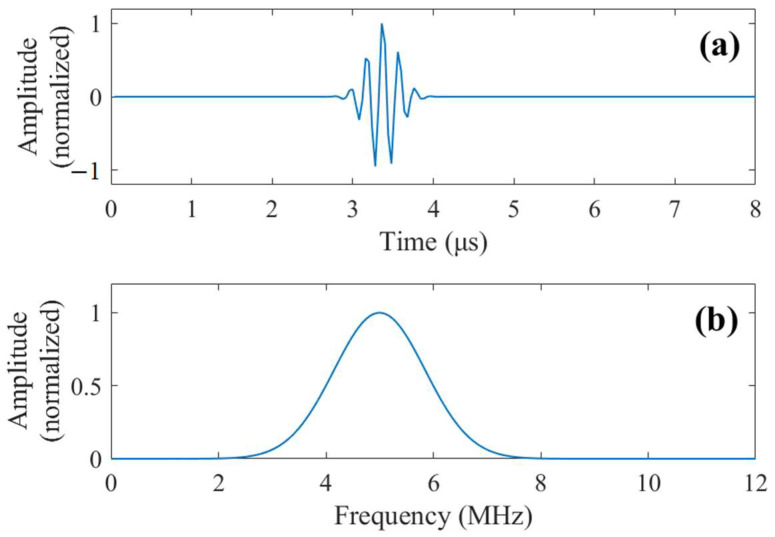
(**a**) Waveform of the transmitted pulse from the transducer. (**b**) Frequency spectrum of the transmitted pulse.

Considering the typical inspection scenario shown in Figure 3, simulation studies are conducted to evaluate the SNR enhancement performance of the proposed method. The ultrasonic transducer used here has a center frequency of 5 MHz and full width at half maximum (FWHM) of 2 MHz. The specimen is a stainless steel block containing two flaws located 60 mm and 72 mm below the surface, respectively. The transmitted pulse is assumed to be a Gabor pulse, which is characterized by:

(19) $$g\left(t\right)=\frac{A}{\sqrt{2\pi }s}exp[-{\left(t-u\right)}^{2}/\left(2s^{2}\right)]exp[i\omega \left(t-u\right)]$$

where A is an energy normalization parameter, s determines the duration of the pulse, u is the time delay of the Gabor pulse, ω is the angular frequency. The frequency spectrum and waveform of the transmitted pulse are given in Figure 4.

**Figure 5:**
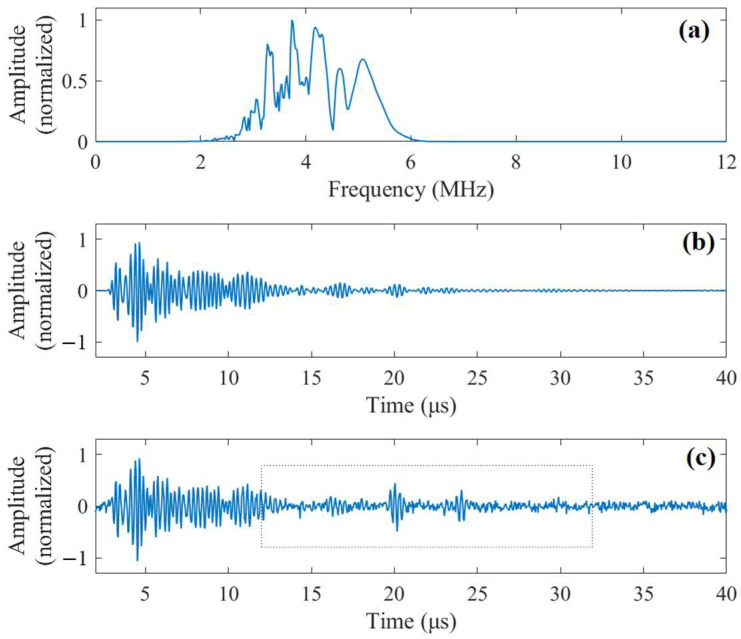
(**a**) Frequency spectrum of simulated grain noise. (**b**) The time-domain waveform of the simulated grain noise. (**c**) The simulated noisy signal.

For the inspection scenario above, the received signal contains two flaw echoes and grain noise backscattered by the grain microstructure of stainless steel as well as white Gaussian noise from the circuits of instruments. Employing the model in [30], the frequency spectrum of grain noise can be expressed as:

(20) $$G\left(\omega \right)=Z\left(\omega \right)\cdot \sum_{j=1}^{K_{g}}\gamma_{j}\frac{\omega^{2}}{d_{j}}exp(-2\alpha d_{j}\omega^{4})exp(-2i\omega d_{j}/v)$$

where $Z\left(\omega \right)$ is the frequency spectrum of the transmitted pulse from the transducer, $K_{g}$ is the number of grain scatterers in the ensonified area, $\gamma_{j}$ is the scattering coefficient of the *j*th grain scatterer which is $d_{j}$ away from the transducer, α is the frequency-dependent attenuation coefficient, v is longitudinal wave velocity in stainless steel. In this study, the parameters are chosen as follows: $K_{g}=2500$, $\alpha =1\times 10^{-28}$, v=6000 m/s. The frequency spectrum of a simulated grain noise by Equation (20) is shown in Figure 5a. Applying inverse Fourier transform to $G\left(\omega \right)$, the time domain waveform of the grain noise $g\left(t\right)$ can be generated as shown in Figure 5b. Similarly, the frequency spectrum of an echo reflected by a flaw at depth $d_{flaw}$ can be expressed as:

(21) $$E\left(\omega \right)=Z\left(\omega \right)\cdot exp(-2\alpha d_{flaw}\omega^{4})\cdot exp(-\frac{2i\omega d_{flaw}}{v})$$

The simulated noisy signal is the superposition of grain noise $g\left(t\right)$ and flaw echoes $e\left(t\right)$ plus some white Gaussian noise. In Figure 5c, the simulated noisy signal is presented. 

To evaluate the SNR enhancement performance of the proposed method, normalized signal-to-noise ratio (NSNR) is introduced which is calculated as [31]:

(22) $$\mathrm{NSNR}=\frac{\sum_{u-l_{p}/2}^{u+l_{p}/2}y{\left(n\right)}^{2}}{\sum_{1}^{N_{s}}y{\left(n\right)}^{2}}$$

where u denotes the time delay of a flaw echo, $l_{p}$ is the width of the flaw echo, and $N_{s}$ is the length of signal $y\left(t\right)$. It can be observed that a noise-free ultrasonic signal has a NSNR=1, and if only noise is present, NSNR approaches 0. Therefore, the enhancement of SNR can be evaluated by comparing NSNR values before and after signal de-noising.

**Figure 6:**
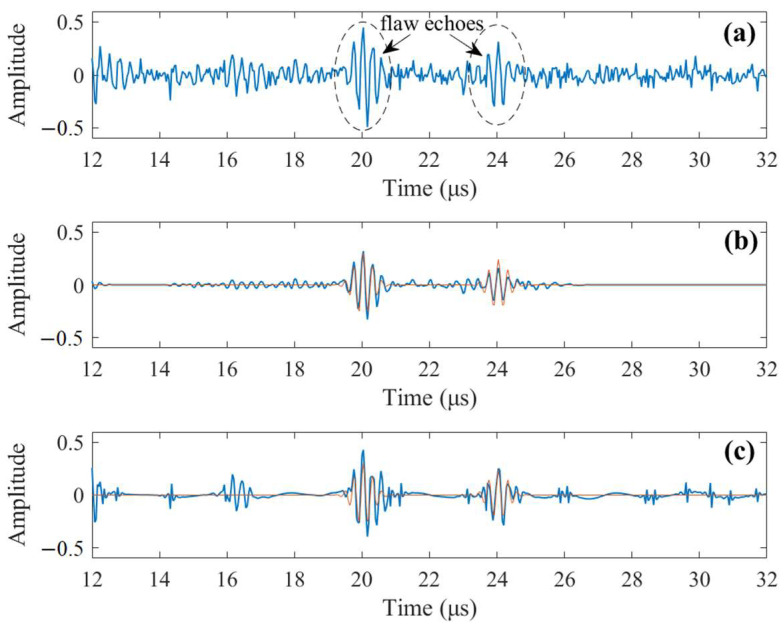
(**a**) The analyzed signal segment containing two flaw echoes, NSNR = 0.5946. (**b**) De-noised signal by our method along with noise-free flaw echoes, NSNR = 0.9488 (blue line: reconstructed signal; red line: original noise-free echoes). (**c**) De-noised signal by DWT (level-4 sym25) along with noise-free flaw echoes, NSNR = 0.7378 (blue line: reconstructed signal; red line: original noise-free echoes).

For the highlighted signal segment in Figure 5c, which contains two flaw echoes as shown in Figure 6a, the NSNR is 0.5946. After signal processing by the proposed method, the de-noised signal is shown in Figure 6b along with the original noise-free flaw echoes. After de-noising, the NSNR increases to 0.9488, meaning that the SNR is significantly increased. Meanwhile, by comparing the de-noised signal and the original noise-free echoes, it can be seen that the waveform of the two echoes can be preserved with high fidelity. For comparison, the four-level discrete wavelet transform (DWT) using the sym25 wavelet proposed in [11] is employed to process the same noisy signal, and the de-noised signal, which has NSNR = 0.7378, is given in Figure 6c. According to [11], after an extensive study, the authors concluded that a four-level sym25 wavelet yields the best result for signal-to-noise ratio enhancement. The comparison clearly demonstrates a better performance of our method. Although the second reconstructed echo by our method has a slightly lower amplitude, the majority of noise is effectively removed. On the contrary, the de-noised signal by four-level sym25 DWT still contains notable residual noise, resulting in a lower NSNR value.

### 4.2. Experimental Signals

**Figure 7:**
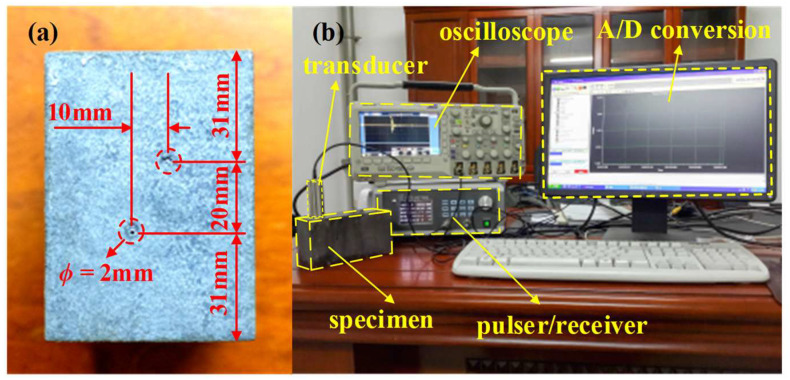
(**a**) A stainless steel specimen with two flaws. (**b**) The setup of the experiment system.

**Figure 8:**
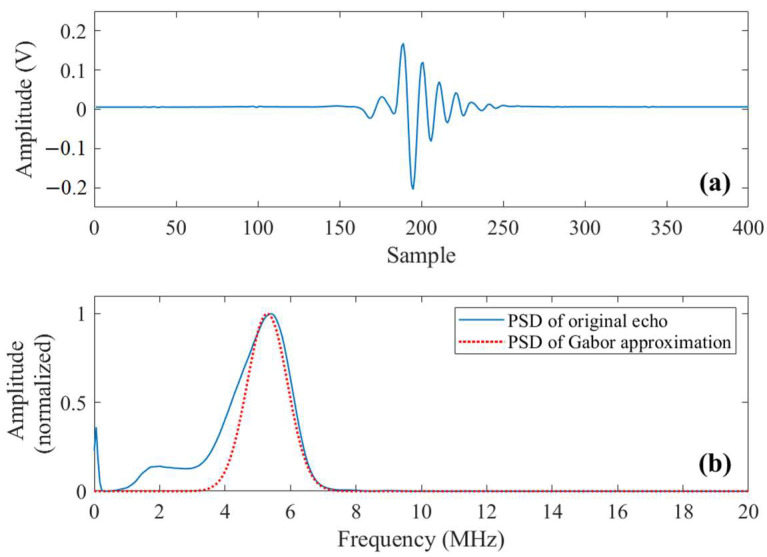
(**a**) The measured pulse transmitted by the transducer. (**b**) Frequency spectrum (F.S.) of the transmitted pulse and its Gaussian fitting.

In this section, the proposed methodology is employed to process signals obtained from ultrasonic inspection of a stainless steel block in the laboratory to demonstrate its effectiveness. The specimen being tested is a heat-treated Type 304 stainless steel block with two fabricated flaws (side drilled holes with a diameter of 2 mm) as shown in Figure 7a. The dimensions of the specimen are 150 mm × 82 mm × 59 mm, and the two holes are about 31 mm and 51 mm below the top surface, respectively. Heat treatment is conducted at a temperature of 1250 °C for 8 h, followed by quenching in a water bath. Empirically, grain sizes around $\bar{D}\approx 120\;\mu m$ will be formed. The ultrasonic DNT system is shown in Figure 7b. The transducer used is type 5P20 made by Shantou Institute of Ultrasonic Instruments Co., Ltd. (Shantou, China), with a center frequency of 5 MHz. The ultrasonic pulse transmitted by the transducer is given in Figure 8a and the frequency spectra of the transmitted pulse is shown in Figure 8b. The ultrasonic pulse/receiver, Type 5072PR, is from Olympus Co., Ltd.(Tokyo, Japan). A-scan signals are obtained in pulse-echo mode and sampled at 50 MHz using DPO2024 oscilloscope produced by Tektronix, Inc. (Beaverton, OR, USA). 

Longitudinal sound velocity in the block is measured first on the time-of-flight principle. The thickness of the specimen is measured ten times using a Vernier caliper, and the average thickness of the specimen is about 81.4 mm. The round-trip travel time of the ultrasonic pulse reflected by the bottom of the specimen is 28.4 μs; therefore, the longitudinal sound velocity equals v=5735 m/s. Now one can notice that the wavelength of the ultrasonic pulse is λ≈1 mm, which means $\lambda /\bar{D}\gg 1$, confirming that the scattering is in the Rayleigh region.

**Figure 9:**
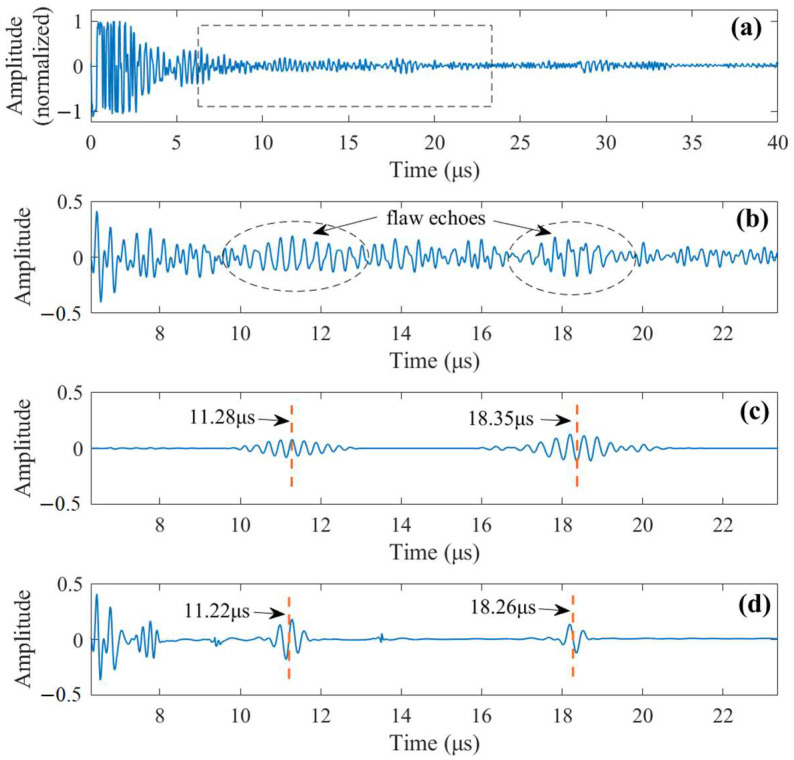
(**a**) A noisy signal measured from the experiment. (**b**) The signal to be analyzed containing two flaw echoes, NSNR = 0.2864. (**c**) The signal de-noised by our method, NSNR = 0.9596. (**d**) The signal de-noised by DWT (4-level sym25), NSNR = 0.2685.

In Figure 9a, an experimental signal obtained when the transducer is positioned between the two flaws so the received signal contains two flaw echoes is presented. The signal segment to be analyzed is highlighted in Figure 9a and shown in Figure 9b, which has an NSNR = 0.2864. By using the proposed method, the de-noised signal is shown in Figure 9c. Notable SNR enhancement is observed, as its NSNR now increases to 0.9596. From the de-noised signal, the time delays of the two flaw echoes are easily identified, which are 11.28 μs and 18.35 μs, respectively. Based on the time-of-flight principle, the depths of the flaws are obtained and compared with their actual values, as tabulated in Table 1. The detection results reveal good detection accuracy. For comparison, the DWT approach is implemented to process the same noisy signal, revealing NSNR = 0.2685. The low value of NSNR is due to the inability of the DWT approach to remove the strong grain noise on the left side. The de-noised signal by the DWT approach (4-level sym25) is shown in Figure 9d. Although the two flaw echoes are successfully revealed and the yielded time delays are in line with our method, the waveform of the two flaw echoes in the de-noised signal is not sound, because theoretically, the duration of the second flaw echo will be longer than the first one due to frequency-dependent attenuation.

## 5. Conclusions

In this work, a novel time-frequency filtering approach is proposed to enhance the ultrasonic inspection performance of stainless steel structures by suppressing noise induced by the grain microstructure of stainless steel; thus, increasing the SNR of the measured signal. Spectral modeling of a flaw echo and grain noise shows that, in the same time frame, the spectrum of a flaw echo and that of grain noise exhibits different characteristics which can be exploited to suppress the energy of noise. As such, the spectral similarity index is introduced, which is used to determine the shape of the Tukey window in the filtering stage and to determine the weighting coefficients in the signal recombination stage. This approach has been validated both in the processing of simulated signals and signals measured from experiments. In both cases, the enhancement of SNR is significant. The high fidelity of the de-noised signal enables accurate flaw depth detection, showing good performance of the proposed method. Moreover, the method is also effective for scenarios where multiple flaws exist.

## Figures and Tables

**Table 1:** Flaw detection results using the proposed method.

	Upper Flaw	Lower Flaw
Detected depth (mm)	32.3	52.6
Actual depth (mm)	31.0	51.0
Absolute error (mm)	1.3	1.6

## Data Availability

Not applicable.
